# Fixed point theorems on multi valued mappings in b-metric spaces

**DOI:** 10.1186/s40064-016-1870-9

**Published:** 2016-02-29

**Authors:** J. Maria Joseph, D. Dayana Roselin, M. Marudai

**Affiliations:** P.G. and Research Department of Mathematics, St. Joseph’s College, Tiruchirappalli, Tamil Nadu 620 002 India; Department of Mathematics, Auxilium College of Arts and Science for Women, Thanjavur, Tamil Nadu 614 602 India; P.G. and Research Department of Mathematics, Bharathidasan University, Tiruchirappalli, Tamil Nadu 620 024 India

**Keywords:** b-Metric space, Multi-valued mappings, Contraction, Fixed point

## Abstract

In this paper, we prove a fixed point theorem and a common fixed point theorem for multi valued mappings in complete b-metric spaces.

## Introduction and preliminaries

Fixed point theory plays one of the important roles in nonlinear analysis. It has been applied in physical sciences, Computing sciences and Engineering. In 1922, Stefan Banach proved a famous fixed point theorem for contractive mappings in complete metric spaces. Later, Czerwik (1993, 1998) has come up with b-metrics which generalized usual metric spaces. After his contribution, many results were presented in $\beta$-generalized weak contractive multifunctions and b-metric spaces (Alikhani et al. 2013; Boriceanu 2009; Mehemet and Kiziltunc 2013). The following definitions will be needed in the sequel:

### **Definition 1**

Nadler (1969) Let *X* and *Y* be nonempty sets. *T* is said to be multi-valued mapping from *X* to *Y* if *T* is a function for *X* to the power set of *Y*. we denote a multi-valued map by:

$$T{: }X \rightarrow 2^Y.$$

### **Definition 2**

Nadler (1969) A point of $x_0\in X$ is said to be a fixed point of the multi-valued mapping *T* if $x_0\in Tx_0$.

### *Example 3*

Joseph (2013) Every single valued mapping can be viewed as a multi-valued mapping. Let $f{: }X \rightarrow Y$ be a single valued mapping. Define $T{: }X \rightarrow 2^Y$ by $Tx=\{f(x)\}$. Note that *T* is a multi-valued mapping iff for each $x\in X, TX\subseteq Y$. Unless otherwise stated we always assume *Tx* is non-empty for each $x,y\in X$.

### **Definition 4**

Banach (1922) Led (*X*, *d*) be a metric space. A map $T{: }X\rightarrow X$ is called contraction if there exists $0\le \lambda <1$ such that $d(Tx,Ty)\le \lambda d(x,y)$, for all $x,y\in X$.

### **Definition 5**

Nadler (1969) Let (*X*, *d*) be a metric space. We define the Hausdorff metric on *CB*(*X*) induced by *d*. That is

$$H(A,B)= max\{\sup _{x\in A}d(x,B),\sup _{y\in B}d(y,A)\}$$

for all $A,B\in CB(X)$, where *CB*(*X*) denotes the family of all nonempty closed and bounded subsets of *X* and $d(x,B)=\inf \{d(x,b):b\in B\}$, for all $x\in X$.

### **Definition 6**

Nadler (1969) Let (*X*, *d*) be a metric space. A map $T{: }X\rightarrow CB(X)$ is said to be multi valued contraction if there exists $0\le \lambda < 1$ such that $H(Tx,Ty)\le \lambda d(x,y)$, for all $x,y\in X$

### **Lemma 7**

Nadler (1969) *If*$A,B \in CB(X)$*and*$a\in A$*, then for each*$\epsilon >0$*, there exists*$b\in B$*such that*$d(a,b) \le H(A,B)+ \epsilon$.

### **Definition 8**

Aydi et al. (2012) Let *X* be a nonempty set and let $s\ge 1$ be a given real number. A function $d{: }X\times X\rightarrow \mathbb {R^+}$ is called a b-metric provide that, for all $x,y,z\in X$,

1. $d(x,y)=0$ if and only if $x=y$
2. $$d(x,y)= d(y,x)$$
3. $$d(x,z)\le s[d(x,y)+d(y,z)]$$.

A pair(*X*, *d*) is called a b-metric space.

### *Example 9*

Boriceanu (2009) The space $l_p(0<p<1)$, $l_p = \{ (x_n{: }\displaystyle \sum\nolimits _{n=1}^\infty |x_n|^p <\infty \}$, together with the function $d{: }l_p\times l_p\rightarrow \mathbb {R^+}$.

### *Example 10*

Boriceanu (2009) The space $L_p(0<p<1)$ for all real function $x(t), t\in [0,1]$ such that $\int _0^1|x(t)|^p dt< \infty$, is *b*-metric space if we take $d(x,y)= (\int _0^1 |x(t)-y(t)|^p dt)^{\frac{1}{p}}$.

### *Example 11*

Aydi et al. (2012) Let $X=\{0,1,2\}$ and $d(2,0)=d(0,2)=m\ge 2$, $d(0,1)=d(1,2)=d(0,1)=d(2,1)=1$ and $d(0,0)=d(1,1)=d(2,2)=0$. Then $d(x,y)\le \frac{m}{2}[d(x,z)+d(z,y)]$ for all $x,y,z\in X$. If $m>2$,the ordinary triangle inequality does not hold.

### **Definition 12**

Boriceanu (2009) Let (*X*, *d*) be a *b*-metric space. Then a sequence $(x_n)$ in *X* is called Cauchy sequence if and only if for all $\epsilon >0$ there exists $n(\epsilon )\in \mathbb {N}$ such that for each $m,n\ge n(\epsilon )$ we have $d(x_n,x_m)< \epsilon$.

### **Definition 13**

Boriceanu (2009) Let be a (*X*, *d*) *b*-metric space. Then a sequence $(x_n)$ in X is called convergent sequence if and only if there exists $x\in X$ such that for all $\epsilon >0$ there exists $n(\epsilon )\in \mathbb {N}$ such that for all $n\ge n(\epsilon )$ we have $d(x_n,x)<\epsilon$. In this case we write $\displaystyle \lim _{n\rightarrow \infty }x_n =x$

Our first result is the following theorem.

## Main results

### **Definition 14**

Let (*X*, *d*) be a *b*-metric space with constant $s\ge 1$. A map $T{: }X\rightarrow CB(X)$ is said to be multi valued generalized contraction if

1 $$\begin{aligned} H(Tx,Ty)&\le a_{1}d(x,Tx)+a_{2}d(y,Ty)+a_{3}d(x,Ty)+ a_{4}d(y,Tx)+a_{5}d(x,y) \\&\quad +a_{6}\dfrac{d(x,Tx)(1+d(x,Tx))}{1+d(x,y)}, \end{aligned}$$

for all $x,y \in X$ and $a_i\ge 0,\quad i=1,2,3,\ldots 6$ with $a_1+a_2+2sa_3+a_4+a_5+a_6 < 1$.

### **Theorem 15**

*Let* (*X*, *d*) *be a completeb*-*metric space with constant*$s\ge 1$. *Let*$T:X\rightarrow CB(X)$*be a multi valued generalized contraction mapping. Then T has a unique fixed point*.

### *Proof*

Fix any $x\in X$. Define $x_0=x$ and let $x_1\in Tx_0$. By Lemma 7, we may choose $x_2\in Tx_1$ such that $d(x_1,x_2)\le H(Tx_0,Tx_1)+(a_1+sa_3+a_5+a_6)$.

Now,

$$\begin{aligned} d(x_1,x_2)& \le H(Tx_0,Tx_1)+(a_1+sa_3+a_5+a_6)\\ &\le a_1d(x_0,Tx_0)+a_2d(x_1,Tx_1)+a_3d(x_0,Tx_1)+a_4d(x_1, Tx_0)\\&\quad +a_5d(x_0, x_1)+ a_6\frac{d(x_0,Tx_0)(1+d(x_0,Tx_0))}{1+d(x_0,x_1)} +(a_1+sa_3+a_5+a_6)\\ d(x_1,x_2)& \le a_1d(x_0,x_1)+a_2d(x_1,x_2)+a_3d(x_0,x_2)+a_4d(x_1,x_1)+a_5d(x_0, x_1)\\ &\quad + a_6d(x_0,x_1)+(a_1+sa_3+a_5+a_6)\\ & \le (a_1+a_5+a_6) d(x_0,x_1) + a_2d(x_1,x_2)+ a_3 s[d(x_0,x_1)+d(x_1,x_2)]\\ &\quad +(a_1+sa_3+a_5+a_6)\\ & \le (a_1+sa_3+a_5+a_6) d(x_0,x_1) + a_2d(x_1,x_2)+ sa_3 d(x_1,x_2) \\&\quad +(a_1+sa_3+a_5+a_6)\\ d(x_1,x_2)& \le \frac{(a_1+sa_3+a_5+a_6)}{1-(a_2+sa_3)} d(x_0,x_1)+\frac{(a_1+sa_3+a_5+a_6)}{1-(a_2+sa_3)}\\ \end{aligned}$$

By Lemma 7, there exist $x_3\in Tx_2$ such that $d(x_2,x_3)\le d(Tx_1,x_2)+\frac{(a_1+sa_3+a_5+a_6)^2}{1-(a_2+sa_3)}$.

Now,

$$\begin{aligned} d(x_2,x_3)\le & {} H(Tx_1,x_2)+\frac{(a_1+sa_3+a_5+a_6)^2}{1-(a_2+sa_3)}\\ \le & {} a_1d(x_1,Tx_1)+a_2d(x_1,Tx_2)+a_3d(x_1,Tx_2)\\&\quad +a_4d(x_2,Tx_1)+a_5d(x_1, x_2)+ a_6d(x_1,x_2)+\frac{(a_1+sa_3+a_5+a_6)^2}{1-(a_2+sa_3)}\\ \le & {} \frac{(a_1+sa_3+a_5+a_6)}{1-(a_2+sa_3)} d(x_1,x_2)+\frac{(a_1+sa_3+a_5+a_6)^2}{(1-(a_2+sa_3))^2}\\ d(x_2,x_3)\le & {} \Big (\frac{(a_1+sa_3+a_5+a_6)}{1-(a_2+sa_3)}\Big )^2 d(x_0,x_1)+2\Big [\frac{(a_1+sa_3+a_5+a_6)}{(1-(a_2+sa_3))}\Big ] ^2\\ \end{aligned}$$

Continuing this process, we obtain by induction a sequence $\{x_n\}$ such that $x_n\in Tx_{n-1}, x_{n+1}\in Tx_n$ such that

$$d(x_n,x_{n+1}) \le \frac{(a_1+sa_3+a_5+a_6)}{1-(a_2+sa_3)} d(x_{n-1},x_n)+\Big [\frac{(a_1+sa_3+a_5+a_6)}{(1-(a_2+sa_3))}\Big ] ^n$$

for all $n\in \mathbb {N}$ and let $k=\frac{(a_1+sa_3+a_5+a_6)}{1-(a_2+sa_3)}$

$$\begin{aligned} d(x_n,x_{n+1})& \le kd(x_{n-1},x_n)+k^n\\ & \le k\Big [kd(x_{n-2},x_{n-1})+k^{n-1}\Big ]+k^n\\ &= k^2d(x_{n-2},x_{n-1})+k k^{n-1}+k^n\\&\vdots&\\ d(x_n,x_{n+1})& \le k^nd(x_0,x_1)+nk^n\\ \end{aligned}$$

Since $k<1, \sum k^n$ and $\sum nk^n$ have same radius of convergence. Then, $\{x_n\}$ is a Cauchy sequence. But (*X*, *d*) is a complete *b*-metric space, it follows that $\{x_n\}_{n=0}^\infty$ is convergent.

$$u=\lim _{n\rightarrow \infty } x_n.$$

Now,

$$\begin{aligned} d(u,Tu)& \le s\Big [d(u,x_{n+1})+d(x_{n+1},Tu)\Big ]\\ d(u,Tu)& \le s\Big [d(u,x_{n+1})+d(Tx_n,Tu)\Big ] \end{aligned}$$

Using (1), we obtain,

$$\begin{aligned} &d(u,Tu) \le s[d(u,x_{n+1})]+s\Big [a_1 d(x_n,Tx_n)+a_2 d(u,Tu)+a_3 d(x_n,Tu)+a_4 d(u,Tx_n)+ a_5 d(x_n,u)+a_6 d(x_n,u)\Big ].\\ &As\quad n\rightarrow \infty ,\\ &d(u,Tu) \le s\Big [a_2 d(u,Tu)+a_3d(u,Tu)\Big ]\\ &\big (1-(a_2s+a_3s)\big ) d(u,Tu) \le 0. \end{aligned}$$

The above inequality is true unless $d(u,Tu)=0$. Thus, $Tu=u$.

Now we show that *u* is the unique fixed point of T. Assume that *v* is another fixed point of *T*. Then we have $Tv=v$ and

$$\begin{aligned} d(u,v)&= d(Tu,Tv)\\ & \le s\big [d(u,Tv)+d(v,Tu)\big ] \end{aligned}$$

we obtain, $d(u,v)\le 2s d(u,v)$. This implies that $u = v$. This completes the proof.$\square$

### **Theorem 16**

*Let*$\left( X,d\right)$*be a complete b-metric space with constant*$\lambda \ge 1$. *Let*$T, S{: }X\rightarrow CB \left( X\right)$*be a multi valued mapping satisfies the condition*:

$$\begin{aligned} H(Tx,Sy)&\le a_{1}d(x,Tx)+a_{2}d(y,Sy)+a_{3}d(x,Sy)+ a_{4}d(y,Tx) +a_{5}d(x,y), \end{aligned}$$

*for all x,y*$$\in$$*X and*$$a_i\ge 0,$$$$i=1,2,\ldots 5,$$*with*$$\left( a_1+a_2\right) \left( \lambda +1\right) + \left( a_3+a_4\right) \left( \lambda ^2+ \lambda \right) +2\lambda a_5 < 2,$$$$a_1+a_2+a_3+a_4+a_5 < 1.$$*Then T and S have a unique common fixed point*.

### *Proof*

Fix any $x\in X.$ Define $x_0 = x$ and let $x_1 \in Tx_0,x_2 \in Sx$ such that $x_{2n+1} = Tx_{2n},x_{2n+2} = Sx_{2n+1},$ By Lemma 7, we may choose $x_2 \in Sx_1$ such that $d \left( x_1,x_2\right) \le H \left( Tx_0,Sx_1\right) +\left( a_1+a_5+\lambda a_3\right)$

2 $$\begin{aligned} d\left( x_1,x_2\right)& \le a_1d\left( x_0,Tx_0\right) +a_2d\left( x_1,Sx_1\right) +a_3d\left( x_0,Sx_1\right) +a_4d\left( x_1,Tx_0\right) \\&\quad + a_5d\left( x_0,x_1\right) +\left( a_1+a_5+\lambda a_3\right) \\& = a_1d\left( x_0,x_1\right) +a_2d\left( x_1,x_2\right) +a_3d\left( x_0,x_2\right) \\&\quad +a_4d\left( x_0,x_1\right) +a_5d\left( x_0,x_1\right) +\left( a_1+a_5+\lambda a_3\right) \\ & \le a_1d\left( x_0,x_1\right) +a_2d\left( x_1,x_2\right) +a_3 \lambda \left[ d\left( x_0,x_1\right) +d\left( x_1,x_2\right) \right] \\&\quad +a_5d\left( x_0,x_1\right) +\left( a_1+a_5+\lambda a_3\right) \\ d\left( x_1,x_2\right)& \le \left( a_1+\lambda a_3+a_5\right) d\left( x_0,x_1\right) +\left( a_2+\lambda a_3\right) d\left( x_1,x_2\right) +\left( a_1+a_5+\lambda a_3\right) \\ d\left( x_1,x_2\right)& \le \dfrac{ \left( a_1+a_5+\lambda a_3\right) }{1- \left( a_2+\lambda a_3\right) }d\left( x_0,x_1\right) + \dfrac{ \left( a_1+a_5+\lambda a_3\right) }{1- \left( a_2+\lambda a_3\right) } \end{aligned}$$

On the other hand and by symmetry,we have

3 $$\begin{aligned} d\left( x_2,x_1\right)&= d\left( Sx_1,Tx_0\right) \\ & \le H\left( Sx_1,Tx_0\right) +\left( a_2+a_5+\lambda a_4\right) \\ & \le a_1d\left( x_1,Sx_1\right) +a_2d\left( x_0,Tx_0\right) +a_3d\left( x_1,Tx_0\right) +a_4d\left( x_0,Sx_1\right) \\&\quad +a_5d\left( x_1,x_0\right) +\left( a_2+a_5+\lambda a_4\right) \\& = a_1d\left( x_1,x_2\right) +a_2d\left( x_0,x_1\right) +a_3d\left( x_1,x_1\right) +a_4d\left( x_0,x_2\right) \\&\quad +a_5d\left( x_0,x_1\right) +\left( a_2+a_5+\lambda a_4\right) \\ & \le a_1d\left( x_1,x_2\right) +a_2d\left( x_0,x_1\right) +a_4\left[ d\left( x_0,x_1\right) +d\left( x_1,x_2\right) \right] +a_5d\left( x_0,x_1\right) \\&\quad +\left( a_2+a_5+\lambda a_4\right) \\& = \left( a_2+a_5+\lambda a_4\right) d\left( x_0,x_1\right) +\left( a_1+\lambda a_4\right) d\left( x_2,x_1\right) \left( a_2+a_5+\lambda a_4\right) \\ d\left( x_2,x_1\right)& \le \dfrac{ \left( a_2+a_5+\lambda a_4\right) }{1- \left( a_1+\lambda a_4\right) }d\left( x_0,x_1\right) + \dfrac{ \left( a_2+a_5+\lambda a_4\right) }{1- \left( a_1+\lambda a_4\right) } \end{aligned}$$

Adding inequalities (2) and (3) , we obtain

$$\begin{aligned} d\left( x_1,x_2\right)&\le \frac{ a_1+a_2+Sa_3+Sa_4+2a_5}{2- \left( a_1+a_2+Sa_3+Sa_4\right) }d\left( x_0,x_1\right) + \frac{ (a_1+a_2+Sa_3+Sa_4+2a_5)}{2- \left( a_1+a_2+Sa_3+Sa_4\right) }\\ where, k&= \frac{ (a_1+a_2+\lambda a_3+\lambda a_4+2a_5}{2- \left( a_1+a_2+\lambda a_3+\lambda a_4\right) } < \frac{1}{\lambda }. \end{aligned}$$

Similarly, it can be shown that, there exists $x_3 \in Tx_2$ such that

$$\begin{aligned} d\left( x_3,x_2\right)&\le H\left( Tx_2,Sx_1\right) + \left( \frac{a_1+a_2+\lambda a_3+\lambda a_4+2a_5}{2- \left( a_1+a_2+\lambda a_3+\lambda a_4\right) }\right) ^2\\&\le k^2 d\left( x_1,x_0\right) +2k^2 \end{aligned}$$

Continuing this process,we obtain by induction a sequence $\left\{ x_n\right\}$ such that $x_{2n+1} \in Tx_{2n},x_{2n+2}\in Sx_{2n+1}$ such that

4 $$\begin{aligned} d\left( x_{2n+1},x_{2n+2}\right)& \le Hd\left( Tx_{2n},Sx_{2n+1}\right) +\left( a_1+a_5+\lambda a_3\right) ^{2n+1} \\& \le a_1 d\left( x_{2n},Tx_{2n}\right) +a_2 d\left( x_{2n+1},Sx_{2n+1}\right) +a_3 d\left( x_{2n},Sx_{2n+1}\right) \\&\quad + a_4 d\left( x_{2n+1},Tx_{2n}\right) +a_5 d\left( x_{2n},x_{2n+1}\right) +\left( a_1+a_5+\lambda a_3\right) ^{2n+1} \\ d\left( x_{2n+1},x_{2n+2}\right)& \le \dfrac{\left( a_1+a_5+\lambda a_3\right) }{1-\left( a_2+ \lambda a_3\right) }d\left( x_{2n},x_{2n+2}\right) + \dfrac{\left( a_1+a_5+\lambda a_3\right) ^{2n+1}}{\left( 1-\left( a_2\lambda a_3\right) \right) ^{2n+1}} \end{aligned}$$

Also,

5 $$\begin{aligned} d\left( x_{2n+2},x_{2n+1}\right)& \le \frac{\left( a_2+a_5+\lambda a_4\right) }{1-\left( a_1+ \lambda a_4\right) }d\left( x_{2n+1},x_{2n}\right) + \frac{\left( a_2+a_5+\lambda a_4\right) ^{2n+1}}{\left( 1-\left( a_2\lambda a_3\right) \right) ^{2n+1}} \end{aligned}$$

From (4) and (5)

$$d\left( x_{2n+1},x_{2n+2}\right) \le kd\left( x_{2n+1},x_{2n}\right) +k^{2n+1}$$

Therefore,

$$\begin{aligned} d\left( x_{n},x_{n+1}\right)&\le \frac{ a_1+a_2+\lambda a_3+\lambda a_4+2a_5}{2- \left( a_1+a_2+\lambda a_3+\lambda a_4\right) }d\left( x_{n-1},x_{n}\right) \\ &\quad +\left( \frac{a_1+a_2+\lambda a_3+\lambda a_4+2a_5}{2- \left( a_1+a_2+\lambda a_3+\lambda a_4\right) }\right) ^n\\ for \quad all \quad&n \in \mathbf N \quad and\quad let\quad k= \frac{ (a_1+a_2+\lambda a_3+\lambda a_4+2a_5}{2- \left( a_1+a_2+\lambda a_3+\lambda a_4\right) }\\ d\left( x_{n},x_{n+1}\right)&\le kd\left( x_{n-1},x_{n}\right) +k^n\\& \le k \left( d\left( x_{n-2},x_{n-1}\right) +k^{n-1} \right) +k^n\\& = k^2 d\left( x_{n-2},x_{n-1}\right) +2k^n\\ &\le \cdots \cdots \cdots \\ & \le k^nd\left( x_0,x_1\right) +nk^n. \end{aligned}$$

Since $0 < k < 1,\sum k^n$ and $\sum nk^n$ have same radius of convergence. Then, $\left\{ x_n\right\}$ is a Cauchy sequence. Since $\left( X,d\right)$ is complete,there exists $z\in X$ such that $x_n \rightarrow z$.

We shall prove that *z* is a common fixed point of T and S.

6 $$\begin{aligned} d\left( z,Tz\right)& \le \lambda \left[ d\left( z,x_{2n+1}\right) +d\left( x_{2n+1},Tz\right) \right] \\ & \le \lambda \left[ d\left( z,x_{2n+1}\right) +H\left( x_{2n+1},Tz\right) \right] \\ d\left( z,Sz\right)& \le \lambda \left[ d\left( z,x_{2n+1}\right) +d\left( x_{2n+1},Sz\right) \right] \\ & \le \lambda \left[ d\left( z,x_{2n+1}\right) +H\left( x_{2n},Sz\right) \right] \end{aligned}$$

7 $$\begin{aligned} H\left( x_{2n},Sz\right)& \le a_1d\left( x_{2n},Tx_{2n}\right) +a_2d\left( z,Sz\right) +a_3d\left( x_{2n},Sz\right) +a_4d\left( z,Tx_{2n}\right) \\&\quad +a_5d\left( x_{2n},z\right) \end{aligned}$$

Using (7) in (6) and letting as $n\rightarrow \infty$, we obtain,

$$\begin{aligned} d(z,Sz)& \le \lambda d(z,z)+ \lambda [a_1d(z,z)+a_2d(z,Sz)+a_3d(z,Sz)+a_4d(z,z) + a_5d(z,z)]\\ &= \lambda [ a_2d(z,Sz)+a_3 d(z,Sz)] \\ & \le \lambda (a_2+a_3) d(z,Sz) \end{aligned}$$

$$[1-\lambda (a_2+ a_3)] d(z,Sz) \le 0.$$

$1-\lambda \left( a_2+a_3\right) \le 0$ and $S\left( z\right)$is closed. Thus, $S\left( z\right) = z$.

Similarly, $$T\left( z\right) =z$$.

We show that *z* is the unique fixed point of S and T. Now,

$$\begin{aligned} d(z,v)& \le H(Tz,Sv)\\ & \le a_1d(z,Tz)+a_2d(v,Sv)+a_3d(z,Sv)+a_4d(v,Tz)+a_5d(z,v)\\ & \le a_3d(z,v)+a_4d(z,v)+a_5d(z,v). \end{aligned}$$

Since $[1-(a_3+a_4+a_5)] > 0, d(z,v)=0.$ Hence, S and T have a unique common fixed point.$\square$

### *Example 17*

Let $X=\mathbf R$. We define $d:X \times X \rightarrow X$ by $d(x,y)= ( \vert x-y \vert )$, for all $x,y \in X$. Then (*X*, *d*) is a complete $b-$ metric space.

Define $T:X \rightarrow CB(X)$ by $Tx = \dfrac{x}{10}$, for all $x,y \in X.$ Then,

$$\begin{aligned} H(Tx,Ty)& = \dfrac{1}{10}d(x,y) \left[ where,\,\, a_{1}=a_{2}=a_{3}=a_{4}=a_{6}=0, a_{5}=\dfrac{1}{10}\right] . \end{aligned}$$

Therefore, $0 \in X$ is the unique fixed point of T.

## Conclusion

Many authors have contributed some fixed point results for a self mappings in b-metric spaces. In this paper, we have proved the existence and uniqueness of fixed point results for a multivalued mappings in b-metric spaces. Our contraction mappings also generalize various known contractions like Hardy Roger contraction in the current literature.
